# Supplementary material for: Effect of Lactobacillus rhamnosus hsryfm 1301 Fermented Milk on Lipid Metabolism Disorders in High-Fat-Diet Rats
Source: Nutrients. 2022 Nov 16;14(22):4850. doi: 10.3390/nu14224850 (PMC9698387; doi:10.3390/nu14224850)
Supplement: Supplementary file 1 [file nutrients-14-04850-s001.zip › nutrients-2018211-supplementary.pdf]

Supplementary Table S1. Positive and negative ion mode differential metabolites

Name	Adduct	VIP	P/M	*p*
Cytosine	(M+H)+	1.339816116	0.695514787	0.009473794
1-Myristoyl-sn-glycero-3-phosphocholine	(M+CH3COO+2H)+	1.091267407	0.649149278	0.011145831
1-Stearoyl-2-hydroxy-sn-glycero-3-phosphocholine	(M-H+2Na)+	4.972169111	0.667820504	0.01224513
N6,N6,N6-Trimethyl-L-lysine	(M+H)+	1.093329942	1.547288823	0.01285837
1-O-(cis-9-Octadecenyl)-2-O-acetyl-sn-glycero-3-phosphocholine	M+	3.197333559	0.758079595	0.021859389
L-Glutamine	(M+H)+	2.217074864	1.103079149	0.022673102
1-Stearoyl-sn-glycerol 3-phosphocholine	(M-H+2Na)+	2.318143529	0.865564779	0.032317076
D-Pipecolinic acid	(M+H)+	4.053378183	0.400970799	0.032603689
Deoxycytidine	(2M+H)+	5.374844793	0.932479056	0.038140827
PC(16:0/16:0)	(M+Na)+	2.58811492	0.889237063	0.041170233
1-Oleoyl-sn-glycero-3-phosphocholine	(M+H)+	10.80834747	0.915386944	0.042017126
1-Stearoyl-2-arachidonoyl-sn-glycerol	(M+H-H2O)+	1.508740183	1.173858521	0.042728028
2-Methylbutyroylcarnitine	M+	3.03718377	0.779696835	0.043132864
D-Mannose	(M+NH4)+	1.681792226	1.25222821	0.045425759
Glycocholic acid	(M+H-2H2O)+	2.557808382	0.391055948	0.053130847
Acetylcarnitine	(M+H)+	2.443767544	0.925848725	0.058770911
Creatinine	(M+H)+	3.821129864	1.074570116	0.06438748
Phosphorylcholine	(M+H)+	1.559224201	1.307381519	0.065979468
Betaine	(M+H)+	1.130706336	1.540890823	0.067373186
1,2-dioleoyl-sn-glycero-3-phosphatidylcholine	(M+H)+	13.79637559	1.505841493	0.069259399
1-Palmitoyllysophosphatidylcholine	(2M+Na)+	1.912558767	0.88140669	0.08246131
Thioetheramide-PC	(M-H+2Na)+	4.300371803	0.750789372	0.085299924
L-Palmitoylcarnitine	(M+H)+	1.305891791	1.247146042	0.085562194
Chenodeoxycholate	(M+H-H2O)+	1.392770832	1.59041223	0.095277565
Pelargonic acid	(M-H)-	2.843002769	1.922379931	7.18666E-05
2-Methylbenzoic acid	(M-H)-	4.107659091	3.606561386	0.001096642
Ethyl glucuronide	(M-H)-	1.682640861	0.365010518	0.002722846
Formylanthranilic acid	(M-H)-	2.343991455	0.475128023	0.002989164
Hippuric acid	(M-H)-	1.543684767	0.706984159	0.003363396
Deoxycytidine	(M+CH3COO)-	1.259819095	0.853399702	0.004132452
L-Threonine	(M-H)-	2.01646812	1.495271562	0.006464953

1-Oleoyl-L-.alpha.-lysophosphatidic acid	(M+Na-2H)-	2.019182307	0.76363831	0.008182123
Glyceric acid	(M-H)-	1.278324606	1.274336439	0.009504966
(4Z,7Z,10Z,13Z,16Z,19Z)-4,7,10,13,1 6,19-Docosahexaenoic acid	(M-H)-	10.31339148	0.826504927	0.013823347
Linoleic acid	(M-H)-	2.122233168	0.73877782	0.014047769
3-Indolepropionic acid	(M-H)-	5.24999386	0.672515125	0.015857421
all cis-(6,9,12)-Linolenic acid	(M-H)-	3.590917277	0.846755706	0.018044575
Tricosanoic acid	(M-H)-	1.199714986	0.857942256	0.021169537
Taurolithocholic acid	(M-H)-	1.41017229	1.426131635	0.022514263
Oleic acid	(M-H)-	7.819825296	0.580783115	0.022761338
L-Serine	(M-H)-	1.664792757	1.271227553	0.024583355
Galactonic acid	(M-H)-	1.354859581	1.556746516	0.045269551
Estradiol valerate	(M-H)-	1.473019159	2.17098543	0.046148924
Anthranilic acid (Vitamin L1)	(M-H)-	1.507210003	2.162736392	0.046354175
L-Tyrosine	(M-H)-	2.147055128	0.736347515	0.051497589
3-Hydroxycapric acid	(M-H)-	1.540591523	0.694714155	0.056259294
L-Lysine	(M-H)-	3.765678036	1.738523008	0.05918829
Chenodeoxycholate	(M-H)-	3.942965233	1.823391527	0.07226951
D-Allose	(M+CH3COO)-	1.342046649	0.855268706	0.081130208
Cortisone acetate	(M-H)-	1.335326717	2.52382252	0.092921992
Glycolithocholic acid	(M-H)-	1.162724441	1.699477213	0.095121605
Phenylacetylglycine	(M-H)-	2.116004321	0.832154302	0.096066271

Supplementary Table S2. Metabolomics KEGG pathway

Pathway_Hierarchy2	Map_ID	Map.Name	cpdName	Test	Test All	Ref	RefAll	Test_per	Ref_per	Over_Under	P value	FDR	richFactor
Membrane transport	map02010	ABC transporters	Deoxycytidine|L-Threonine|L-Serine|L-Lysine|D-Allose|L-Glutamine|D-Mannose|Betaine	8	32	128	3389	25	3.776925	over	1.63E-05	0.0016953	0.0625
Digestive system	map04974	Protein digestion and absorption	L-Threonine|L-Serine|L-Tyrosine|L-Lysine|L-Glutamine	5	32	47	3389	15.625	1.38684	over	6.28E-05	0.0031181	0.106383
Translation	map00970	Aminoacyl-tRNA biosynthesis	L-Threonine|L-Serine|L-Tyrosine|L-Lysine|L-Glutamine	5	32	52	3389	15.625	1.534376	over	0.000103	0.0031181	0.096154
Cancers: Overview	map05231	Choline metabolism in cancer	PC(16:0/16:0)|1-Stearoyl-2-arachidonoyl-sn-glycerol|Phosphorylcholine	3	32	11	3389	9.375	0.32458	over	0.00012	0.0031181	0.272727
Amino acid metabolism	map00260	Glycine, serine and	L-Threonine|Glyceric acid|L-	4	32	50	3389	12.5	1.475361	over	0.001112	0.0219376	0.08

		threonine metabolism	Serine|Betaine										
Endocrine system	map04916	Melanogenesis	L-Tyrosine|1-Stearoyl-2-arachidonoyl-sn-glycerol	2	32	6	3389	6.25	0.177043	over	0.001266	0.0219376	0.333333
Lipid metabolism	map01040	Biosynthesis of unsaturated fatty acids	(4Z,7Z,10Z,13Z,16Z,19Z)-4,7,10,13,16,19-Docosahexaenoic acid|Linoleic acid|all cis-(6,9,12)-Linolenic acid|Oleic acid	4	32	54	3389	12.5	1.59339	over	0.001488	0.0221033	0.074074
Lipid metabolism	map00591	Linoleic acid metabolism	Linoleic acid|all cis-(6,9,12)-Linolenic acid|PC(16:0/16:0)	3	32	28	3389	9.375	0.826202	over	0.002135	0.024605	0.107143
Nervous system	map04724	Glutamatergic synapse	L-Glutamine|1-Stearoyl-2-arachidonoyl-sn-glycerol	2	32	8	3389	6.25	0.236058	over	0.002335	0.024605	0.25
Digestive system	map04978	Mineral absorption	L-Threonine|L-Serine|L-Glutamine	3	32	29	3389	9.375	0.85571	over	0.002366	0.024605	0.103448

Cancers: Overview	map05230	Central carbon metabolism in cancer	L-Serine|L-Tyrosine|L-Glutamine	3	32	37	3389	9.375	1.091767	over	0.00478	0.0451891	0.081081
Nervous system	map04728	Dopaminergic synapse	L-Tyrosine|1-Stearoyl-2-arachidonoyl-sn-glycerol	2	32	12	3389	6.25	0.354087	over	0.005375	0.0465836	0.166667
Signal transduction	map04071	Sphingolipid signaling pathway	L-Serine|1-Stearoyl-2-arachidonoyl-sn-glycerol	2	32	16	3389	6.25	0.472116	over	0.009545	0.076363	0.125
Nervous system	map04723	Retrograde endocannabinoid signaling	PC(16:0/16:0)|1-Stearoyl-2-arachidonoyl-sn-glycerol	2	32	19	3389	6.25	0.560637	over	0.013365	0.0992793	0.105263
Endocrine and metabolic diseases	map04931	Insulin resistance	1-Stearoyl-2-arachidonoyl-sn-glycerol|Acetylcarnitine	2	32	21	3389	6.25	0.619652	over	0.016221	0.1124663	0.095238
Carbohydrate metabolism	map00630	Glyoxylate and dicarboxylate metabolism	Glyceric acid|L-Serine|L-Glutamine	3	32	61	3389	9.375	1.799941	over	0.018996	0.1234722	0.04918
Nucleotide metabolism	map00240	Pyrimidine metabolism	Deoxycytidine|Cytosine|L-Glutamine	3	32	66	3389	9.375	1.947477	over	0.023397	0.1431336	0.045455
Signal transduction	map04064	NF-kappa B signaling pathway	1-Stearoyl-2-arachidonoyl-sn-glycerol	1	32	3	3389	3.125	0.088522	over	0.028069	0.1549763	0.333333
Amino acid metabolism	map00360	Phenylalanine metabolism	Hippuric acid|L-	3	32	72	3389	9.375	2.124521	over	0.029354	0.1549763	0.041667

			Tyrosine|Phenylacetylglycine										
Signal transduction	map04012	ErbB signaling pathway	1-Stearoyl-2-arachidonoyl-sn-glycerol	1	32	4	3389	3.125	0.118029	over	0.037254	0.1549763	0.25
Transport and catabolism	map04142	Lysosome	D-Mannose	1	32	4	3389	3.125	0.118029	over	0.037254	0.1549763	0.25
Immune system	map04650	Natural killer cell mediated cytotoxicity	1-Stearoyl-2-arachidonoyl-sn-glycerol	1	32	4	3389	3.125	0.118029	over	0.037254	0.1549763	0.25
Immune system	map04660	T cell receptor signaling pathway	1-Stearoyl-2-arachidonoyl-sn-glycerol	1	32	4	3389	3.125	0.118029	over	0.037254	0.1549763	0.25
Immune system	map04662	B cell receptor signaling pathway	1-Stearoyl-2-arachidonoyl-sn-glycerol	1	32	4	3389	3.125	0.118029	over	0.037254	0.1549763	0.25
Cancers: Specific types	map05214	Glioma	1-Stearoyl-2-arachidonoyl-sn-glycerol	1	32	4	3389	3.125	0.118029	over	0.037254	0.1549763	0.25
Amino acid metabolism	map00400	Phenylalanine, tyrosine and tryptophan biosynthesis	Anthranilic acid (Vitamin L1)|L-Tyrosine	2	32	35	3389	6.25	1.032753	over	0.042351	0.1606981	0.057143
Signal transduction	map04010	MAPK signaling pathway	1-Stearoyl-2-arachidonoyl-sn-glycerol	1	32	5	3389	3.125	0.147536	over	0.046355	0.1606981	0.2
Signal transduction	map04015	Rap1 signaling pathway	1-Stearoyl-2-arachidonoyl-sn-glycerol	1	32	5	3389	3.125	0.147536	over	0.046355	0.1606981	0.2
Immune system	map04062	Chemokine signaling pathway	1-Stearoyl-2-arachidonoyl-sn-glycerol	1	32	5	3389	3.125	0.147536	over	0.046355	0.1606981	0.2

Nervous system	map04722	Neurotrophin signaling pathway	1-Stearoyl-2-arachidonoyl-sn-glycerol	1	32	5	3389	3.125	0.147536	over	0.046355	0.1606981	0.2
Signal transduction	map04370	VEGF signaling pathway	1-Stearoyl-2-arachidonoyl-sn-glycerol	1	32	6	3389	3.125	0.177043	over	0.055373	0.1745093	0.166667
Endocrine system	map04912	GnRH signaling pathway	1-Stearoyl-2-arachidonoyl-sn-glycerol	1	32	6	3389	3.125	0.177043	over	0.055373	0.1745093	0.166667
Cancers: Specific types	map05223	Non-small cell lung cancer	1-Stearoyl-2-arachidonoyl-sn-glycerol	1	32	6	3389	3.125	0.177043	over	0.055373	0.1745093	0.166667
Signal transduction	map04014	Ras signaling pathway	1-Stearoyl-2-arachidonoyl-sn-glycerol	1	32	7	3389	3.125	0.206551	over	0.064308	0.1779436	0.142857
Nervous system	map04720	Long-term potentiation	1-Stearoyl-2-arachidonoyl-sn-glycerol	1	32	7	3389	3.125	0.206551	over	0.064308	0.1779436	0.142857
Endocrine system	map04920	Adipocytokine signaling pathway	1-Stearoyl-2-arachidonoyl-sn-glycerol	1	32	7	3389	3.125	0.206551	over	0.064308	0.1779436	0.142857
Carbohydrate metabolism	map00052	Galactose metabolism	Galactonic acid|D-Mannose	2	32	46	3389	6.25	1.357333	over	0.069118	0.1779436	0.043478
Lipid metabolism	map00120	Primary bile acid biosynthesis	Chenodeoxycholate|Glycocholic acid	2	32	47	3389	6.25	1.38684	over	0.071774	0.1779436	0.042553
Immune system	map04666	Fc gamma R-mediated phagocytosis	1-Stearoyl-2-arachidonoyl-sn-glycerol	1	32	8	3389	3.125	0.236058	over	0.073162	0.1779436	0.125
Substance dependence	map05030	Cocaine addiction	L-Tyrosine	1	32	8	3389	3.125	0.236058	over	0.073162	0.1779436	0.125

Nervous system	map04727	GABAergic synapse	L-Glutamine	1	32	9	3389	3.125	0.265565	over	0.081934	0.1779436	0.111111
Nervous system	map04730	Long-term depression	1-Stearoyl-2-arachidonoyl-sn-glycerol	1	32	9	3389	3.125	0.265565	over	0.081934	0.1779436	0.111111
Endocrine system	map04915	Estrogen signaling pathway	1-Stearoyl-2-arachidonoyl-sn-glycerol	1	32	9	3389	3.125	0.265565	over	0.081934	0.1779436	0.111111
Excretory system	map04961	Endocrine and other factor-regulated calcium reabsorption	1-Stearoyl-2-arachidonoyl-sn-glycerol	1	32	9	3389	3.125	0.265565	over	0.081934	0.1779436	0.111111
Amino acid metabolism	map00310	Lysine degradation	L-Lysine丨N6,N6,N6-Trimethyl-L-lysine	2	32	52	3389	6.25	1.534376	over	0.085539	0.1779436	0.038462
Lipid metabolism	map00564	Glycerophospholipid metabolism	PC(16:0/16:0)丨Phosphorylcholine	2	32	52	3389	6.25	1.534376	over	0.085539	0.1779436	0.038462
Signal transduction	map04020	Calcium signaling pathway	1-Stearoyl-2-arachidonoyl-sn-glycerol	1	32	10	3389	3.125	0.295072	over	0.090626	0.1779436	0.1
Endocrine and metabolic diseases	map04933	AGE-RAGE signaling pathway in diabetic complications	1-Stearoyl-2-arachidonoyl-sn-glycerol	1	32	10	3389	3.125	0.295072	over	0.090626	0.1779436	0.1
Substance dependence	map05031	Amphetamine addiction	L-Tyrosine	1	32	10	3389	3.125	0.295072	over	0.090626	0.1779436	0.1

Substance dependence	map05034	Alcoholism	L-Tyrosine	1	32	10	3389	3.125	0.295072	over	0.090626	0.1779436	0.1
Infectious diseases: Parasitic	map05143	African trypanosomiasis	1-Stearoyl-2-arachidonoyl-sn-glycerol	1	32	10	3389	3.125	0.295072	over	0.090626	0.1779436	0.1
Carbohydrate metabolism	map00051	Fructose and mannose metabolism	D-Allose|D-Mannose	2	32	54	3389	6.25	1.59339	over	0.091256	0.1779436	0.037037
Circulatory system	map04261	Adrenergic signaling in cardiomyocytes	1-Stearoyl-2-arachidonoyl-sn-glycerol	1	32	11	3389	3.125	0.32458	over	0.099238	0.1779436	0.090909
Cellular community	map04540	Gap junction	1-Stearoyl-2-arachidonoyl-sn-glycerol	1	32	11	3389	3.125	0.32458	over	0.099238	0.1779436	0.090909
Immune system	map04664	Fc epsilon RI signaling pathway	1-Stearoyl-2-arachidonoyl-sn-glycerol	1	32	11	3389	3.125	0.32458	over	0.099238	0.1779436	0.090909
Environmental adaptation	map04713	Circadian entrainment	1-Stearoyl-2-arachidonoyl-sn-glycerol	1	32	11	3389	3.125	0.32458	over	0.099238	0.1779436	0.090909
Endocrine system	map04917	Prolactin signaling pathway	L-Tyrosine	1	32	11	3389	3.125	0.32458	over	0.099238	0.1779436	0.090909
Endocrine system	map04919	Thyroid hormone signaling pathway	1-Stearoyl-2-arachidonoyl-sn-glycerol	1	32	11	3389	3.125	0.32458	over	0.099238	0.1779436	0.090909
Metabolism of other amino acids	map00471	D-Glutamine and D-glutamate metabolism	L-Glutamine	1	32	12	3389	3.125	0.354087	over	0.107771	0.1859606	0.083333

Nervous system	map04725	Cholinergic synapse	1-Stearoyl-2-arachidonoyl-sn-glycerol	1	32	12	3389	3.125	0.354087	over	0.107771	0.1859606	0.083333
Signal transduction	map04072	Phospholipase D signaling pathway	1-Stearoyl-2-arachidonoyl-sn-glycerol	1	32	13	3389	3.125	0.383594	over	0.116225	0.1859606	0.076923
Endocrine system	map04911	Insulin secretion	1-Stearoyl-2-arachidonoyl-sn-glycerol	1	32	13	3389	3.125	0.383594	over	0.116225	0.1859606	0.076923
Endocrine system	map04921	Oxytocin signaling pathway	1-Stearoyl-2-arachidonoyl-sn-glycerol	1	32	13	3389	3.125	0.383594	over	0.116225	0.1859606	0.076923
Digestive system	map04975	Fat digestion and absorption	1-Stearoyl-2-arachidonoyl-sn-glycerol	1	32	13	3389	3.125	0.383594	over	0.116225	0.1859606	0.076923
Infectious diseases: Parasitic	map05146	Amoebiasis	1-Stearoyl-2-arachidonoyl-sn-glycerol	1	32	13	3389	3.125	0.383594	over	0.116225	0.1859606	0.076923
Digestive system	map04971	Gastric acid secretion	1-Stearoyl-2-arachidonoyl-sn-glycerol	1	32	14	3389	3.125	0.413101	over	0.124602	0.1963432	0.071429
Signal transduction	map04066	HIF-1 signaling pathway	1-Stearoyl-2-arachidonoyl-sn-glycerol	1	32	15	3389	3.125	0.442608	over	0.132902	0.1974551	0.066667
Immune system	map04611	Platelet activation	1-Stearoyl-2-arachidonoyl-sn-glycerol	1	32	15	3389	3.125	0.442608	over	0.132902	0.1974551	0.066667
Endocrine system	map04923	Regulation of lipolysis in adipocytes	1-Stearoyl-2-arachidonoyl-sn-glycerol	1	32	15	3389	3.125	0.442608	over	0.132902	0.1974551	0.066667
Neurodegenerative diseases	map05012	Parkinson disease	L-Tyrosine	1	32	15	3389	3.125	0.442608	over	0.132902	0.1974551	0.066667

Excretory system	map04964	Proximal tubule bicarbonate reclamation	L-Glutamine	1	32	17	3389	3.125	0.501623	over	0.149274	0.212665	0.058824
Digestive system	map04970	Salivary secretion	1-Stearoyl-2-arachidonoyl-sn-glycerol	1	32	17	3389	3.125	0.501623	over	0.149274	0.212665	0.058824
Digestive system	map04972	Pancreatic secretion	1-Stearoyl-2-arachidonoyl-sn-glycerol	1	32	17	3389	3.125	0.501623	over	0.149274	0.212665	0.058824
Energy metabolism	map00910	Nitrogen metabolism	L-Glutamine	1	32	19	3389	3.125	0.560637	over	0.165347	0.2323794	0.052632
Amino acid metabolism	map00380	Tryptophan metabolism	Formylanthranilic acid丨Anthranilic acid (Vitamin L1)	2	32	81	3389	6.25	2.390086	over	0.177137	0.2456306	0.024691
Endocrine system	map04918	Thyroid hormone synthesis	1-Stearoyl-2-arachidonoyl-sn-glycerol	1	32	21	3389	3.125	0.619652	over	0.181125	0.2478552	0.047619
Amino acid metabolism	map00220	Arginine biosynthesis	L-Glutamine	1	32	23	3389	3.125	0.678666	over	0.196614	0.2621518	0.043478
Amino acid metabolism	map00290	Valine, leucine and isoleucine biosynthesis	L-Threonine	1	32	23	3389	3.125	0.678666	over	0.196614	0.2621518	0.043478
Endocrine system	map04925	Aldosterone synthesis and secretion	1-Stearoyl-2-arachidonoyl-sn-glycerol	1	32	24	3389	3.125	0.708174	over	0.204252	0.2688881	0.041667
Lipid metabolism	map00600	Sphingolipid metabolism	L-Serine	1	32	25	3389	3.125	0.737681	over	0.211819	0.2753645	0.04

Circulatory system	map04270	Vascular smooth muscle contraction	1-Stearoyl-2-arachidonoyl-sn-glycerol	1	32	26	3389	3.125	0.767188	over	0.219316	0.2815914	0.038462
Digestive system	map04973	Carbohydrate digestion and absorption	1-Stearoyl-2-arachidonoyl-sn-glycerol	1	32	27	3389	3.125	0.796695	over	0.226745	0.284114	0.037037
Cancers: Overview	map05200	Pathways in cancer	1-Stearoyl-2-arachidonoyl-sn-glycerol	1	32	27	3389	3.125	0.796695	over	0.226745	0.284114	0.037037
Amino acid metabolism	map00250	Alanine, aspartate and glutamate metabolism	L-Glutamine	1	32	28	3389	3.125	0.826202	over	0.234105	0.2864341	0.035714
Metabolism of cofactors and vitamins	map00780	Biotin metabolism	L-Lysine	1	32	28	3389	3.125	0.826202	over	0.234105	0.2864341	0.035714
Metabolism of cofactors and vitamins	map00730	Thiamine metabolism	L-Tyrosine	1	32	31	3389	3.125	0.914724	over	0.25578	0.3093151	0.032258
Energy metabolism	map00920	Sulfur metabolism	L-Serine	1	32	32	3389	3.125	0.944231	over	0.262872	0.3142375	0.03125
Carbohydrate metabolism	map00030	Pentose phosphate pathway	Glyceric acid	1	32	35	3389	3.125	1.032753	over	0.283757	0.3315815	0.028571
Lipid metabolism	map00561	Glycerolipid metabolism	Glyceric acid	1	32	35	3389	3.125	1.032753	over	0.283757	0.3315815	0.028571
Sensory system	map04750	Inflammatory mediator regulation of TRP channels	1-Stearoyl-2-arachidonoyl-sn-glycerol	1	32	36	3389	3.125	1.06226	over	0.290591	0.3357939	0.027778

Signal transduction	map04024	cAMP signaling pathway	1-Stearoyl-2-arachidonoyl-sn-glycerol	1	32	40	3389	3.125	1.180289	over	0.317299	0.3626274	0.025
Lipid metabolism	map00592	alpha-Linolenic acid metabolism	PC(16:0/16:0)	1	32	42	3389	3.125	1.239304	over	0.330285	0.369351	0.02381
Nervous system	map04726	Serotonergic synapse	1-Stearoyl-2-arachidonoyl-sn-glycerol	1	32	42	3389	3.125	1.239304	over	0.330285	0.369351	0.02381
Lipid metabolism	map00061	Fatty acid biosynthesis	Oleic acid	1	32	50	3389	3.125	1.475361	over	0.379878	0.415866	0.02
Lipid metabolism	map00071	Fatty acid degradation	L-Palmitoylcarnitine	1	32	50	3389	3.125	1.475361	over	0.379878	0.415866	0.02
Amino acid metabolism	map00270	Cysteine and methionine metabolism	L-Serine	1	32	62	3389	3.125	1.829448	over	0.447659	0.4849637	0.016129
Digestive system	map04976	Bile secretion	Chenodeoxycholate|Glycocholic acid	2	32	175	3389	6.25	5.163765	over	0.498234	0.5341895	0.011429
Lipid metabolism	map00590	Arachidonic acid metabolism	PC(16:0/16:0)	1	32	75	3389	3.125	2.213042	over	0.512985	0.5443922	0.013333
Amino acid metabolism	map00330	Arginine and proline metabolism	Creatinine	1	32	77	3389	3.125	2.272057	over	0.522346	0.5480397	0.012987
Amino acid metabolism	map00350	Tyrosine metabolism	L-Tyrosine	1	32	78	3389	3.125	2.301564	over	0.526961	0.5480397	0.012821
Metabolism of cofactors and vitamins	map00130	Ubiquinone and other terpenoid-quinone biosynthesis	L-Tyrosine	1	32	90	3389	3.125	2.655651	over	0.579081	0.5962811	0.011111

Nucleotide metabolism	map00230	Purine metabolism	L-Glutamine	1	32	92	3389	3.125	2.714665	over	0.587208	0.5987219	0.01087
Carbohydrate metabolism	map00520	Amino sugar and nucleotide sugar metabolism	D-Mannose	1	32	108	3389	3.125	3.186781	under	0.646976	0.6532578	0.009259
Metabolism of cofactors and vitamins	map00860	Porphyrin and chlorophyll metabolism	L-Threonine	1	32	139	3389	3.125	4.101505	under	0.739839	0.7398394	0.007194

Supplementary Table S3. Significantly different genes

Gene ID	P	M	log2FoldChange	*P* value	padj	Symbol	Description
Novel.15123	15.57	0.35	5.4075	0.000321	0.088172	-	-
ENSRNOG00000000556	6.69	0	5.1509	0.001848	0.29463	*Nodal*	nodal growth differentiation factor [Source:RGD Symbol;Acc:1305994]
ENSRNOG00000049289	11.39	0.31	4.9578	0.001388	0.24269	*Robo4*	roundabout guidance receptor 4 [Source:RGD Symbol;Acc:727947]
ENSRNOG00000011542	74.14	2.76	4.7556	1.57E-05	0.013701	*Apopt1*	apoptogenic 1, mitochondrial [Source:RGD Symbol;Acc:1304719]
Novel.9837	4914.25	183.34	4.7443	0.000266	0.078327	-	-
Novel.14713	17.62	0.71	4.6801	0.000145	0.058098	-	-
ENSRNOG00000030222	8.3	0.36	4.5002	0.003029	0.38096	*Tcaf2*	TRPM8 channel-associated factor 2 [Source:RGD Symbol;Acc:6487302]
ENSRNOG00000037118	15.27	0.7	4.4832	0.000307	0.085341	*AABR07012779.1*	-
ENSRNOG00000055620	7.08	0.31	4.2684	0.009349	0.73117	*AABR07065768.3*	-
ENSRNOG00000058552	43.72	2.31	4.2172	1.21E-07	0.000316	*AABR07051733.3*	-
Novel.519	3.21	0	4.1007	0.036978	0.99978	-	-
ENSRNOG00000045973	6.11	0.31	4.0555	0.020425	0.99978	*Gzmbl2*	Granzyme B-like 2 [Source:RGD Symbol;Acc:2320097]
ENSRNOG00000050561	23.29	1.62	3.7988	4.44E-05	0.030917	*AABR07065821.1*	-
ENSRNOG00000019346	18.16	1.43	3.7228	0.000305	0.085341	*Tmco3*	transmembrane and coiled-coil domains 3 [Source:RGD Symbol;Acc:1306586]
ENSRNOG00000055375	8.73	0.67	3.6827	0.008666	0.70863	*AABR07065837.1*	-
Novel.15329	8.19	0.62	3.6393	0.00915	0.72648	-	-
ENSRNOG00000047876	4.37	0.35	3.5702	0.042076	0.99978	*LOC689986*	hypothetical protein LOC689986 [Source:RGD Symbol;Acc:1586315]
ENSRNOG0000000175	16.15	1.37	3.5611	0.000786	0.16587	*LOC100911374*	UBX domain-containing protein 7-like

6							[Source:RGD Symbol;Acc:6493076]
ENSRNOG00000018736	86.82	7.94	3.4563	0.000373	0.098571	*Pnpla2*	patatin-like phospholipase domain-containing protein 2-like [Source:RGD Symbol;Acc:6492173]
ENSRNOG00000048118	35.72	3.42	3.3871	0.020274	0.99978	*LOC108348276*	ankyrin repeat family A protein 2 [Source:RGD Symbol;Acc:11492057]
ENSRNOG00000001746	83.68	9.2	3.1868	0.002433	0.35255	*Ncbp2*	nuclear cap binding protein subunit 2 [Source:RGD Symbol;Acc:1596188]
ENSRNOG00000036882	6.09	0.66	3.1822	0.026247	0.99978	*Fbxo47*	F-box protein 47 [Source:RGD Symbol;Acc:1559709]
ENSRNOG00000000590	5.5	0.66	3.0338	0.038604	0.99978	*Naglt1*	Na+ dependent glucose transporter 1 [Source:RGD Symbol;Acc:631438]
Novel.14174	15.19	1.97	2.917	0.001179	0.22857	-	-
ENSRNOG00000051002	26.27	3.62	2.8389	0.002767	0.36996	*LOC108348139*	class II histocompatibility antigen, M beta 1 chain [Source:RGD Symbol;Acc:11469141]
ENSRNOG00000025377	6.81	0.96	2.7704	0.029226	0.99978	*AABR07007758.1*	ankyrin repeat and death domain containing 1B [Source:NCBI gene;Acc:100359662]
ENSRNOG00000003054	13.92	2.06	2.7583	0.018756	0.99978	*AABR07037009.1*	peripheral plasma membrane protein CASK-like [Source:NCBI gene;Acc:100910506]
ENSRNOG00000053812	559.91	85.1	2.7175	0.003564	0.44035	*LOC103689947*	selenium-binding protein 1 [Source:RGD Symbol;Acc:9292945]
ENSRNOG00000046324	9.09	1.44	2.6997	0.02844	0.99978	*AABR07051726.1*	-
ENSRNOG00000059121	6.04	1.02	2.5732	0.04292	0.99978	*AABR07065714.1*	-
ENSRNOG00000046308	124.53	21.29	2.5488	0.001636	0.26476	*LOC100910308*	multifunctional protein ADE2-like [Source:RGD Symbol;Acc:6499293]
ENSRNOG00000050407	25.62	4.47	2.5359	0.002748	0.36996	*Mfsd14a*	major facilitator superfamily domain containing 14A [Source:RGD Symbol;Acc:9294064]
Novel.4651	22.41	4.07	2.4548	0.002202	0.33324	-	-
ENSRNOG00000054334	14.03	2.67	2.3843	0.007164	0.64754	*Abhd10*	abhydrolase domain containing 10 [Source:RGD Symbol;Acc:1308084]
ENSRNOG00000016173	19684.22	3794.13	2.3752	0.002604	0.35901	*Cyp1a2*	cytochrome P450, family 1, subfamily a, polypeptide 2 [Source:RGD Symbol;Acc:2459]

ENSRNOG00000045790	17.68	3.42	2.3703	0.005015	0.54975	*Fam50a*	family with sequence similarity 50, member A [Source:RGD Symbol;Acc:1560964]
ENSRNOG00000054489	311.18	60.76	2.3572	0.04903	0.99978	*AABR07042821.1*	-
ENSRNOG00000046094	122.44	24.24	2.3389	0.00835	0.69737	*Cd151*	CD151 molecule (Raph blood group) [Source:RGD Symbol;Acc:621290]
ENSRNOG00000006957	6.77	1.33	2.3315	0.042991	0.99978	*Gria4*	glutamate ionotropic receptor AMPA type subunit 4 [Source:RGD Symbol;Acc:61863]
Novel.2257	8.26	1.74	2.2624	0.033978	0.99978	-	-
ENSRNOG00000039543	13.24	2.86	2.2499	0.010557	0.7845	*Rnase11*	ribonuclease A family member 11 [Source:RGD Symbol;Acc:1359723]
ENSRNOG00000001854	7.99	1.68	2.2458	0.03234	0.99978	*Tmtc1*	transmembrane and tetratricopeptide repeat containing 1 [Source:RGD Symbol;Acc:1564868]
ENSRNOG00000049009	15.46	3.22	2.2325	0.006444	0.6317	*AABR07051532.1*	-
ENSRNOG00000047571	76.57	16.87	2.1716	1.71E-06	0.002376	*RGD1563231*	similar to immunoglobulin kappa-chain VK-1 [Source:RGD Symbol;Acc:1563231]
ENSRNOG00000057522	84.73	19.14	2.1488	0.043763	0.99978	*Retreg1*	reticulophagy regulator 1 [Source:RGD Symbol;Acc:1565003]
ENSRNOG00000014870	137.6	32.54	2.0744	4.08E-08	0.000122	*Slc13a5*	solute carrier family 13 member 5 [Source:RGD Symbol;Acc:631374]
ENSRNOG00000047657	379.2	90.47	2.0685	9.08E-14	6.32E-10	*C4a*	complement C4A [Source:RGD Symbol;Acc:620005]
ENSRNOG00000012862	13.59	3.39	1.9978	0.019826	0.99978	*Spsb4*	splA/ryanodine receptor domain and SOCS box containing 4 [Source:RGD Symbol;Acc:1562307]
ENSRNOG00000053147	19.21	4.98	1.963	0.036223	0.99978	*AC141521.1*	-
ENSRNOG00000007663	355.89	97.68	1.8653	0.026681	0.99978	*LOC100911372*	40S ribosomal protein S6-like [Source:RGD Symbol;Acc:6493116]
ENSRNOG00000027079	17.94	4.94	1.8424	0.0189	0.99978	*Impad1*	inositol monophosphatase domain containing 1 [Source:RGD Symbol;Acc:1306455]
ENSRNOG00000046068	17.71	5.32	1.7367	0.028643	0.99978	*AABR07027088.1*	-

ENSRNOG00000033261	10.02	2.97	1.7361	0.046357	0.99978	*Fam107a*	family with sequence similarity 107, member A [Source:RGD Symbol;Acc:1306327]
ENSRNOG00000029988	34.38	10.5	1.7286	0.013501	0.88299	*AABR07007675.1*	-
ENSRNOG00000032232	84.45	26.16	1.6981	0.000102	0.047496	*Snrpg*	small nuclear ribonucleoprotein polypeptide G [Source:RGD Symbol;Acc:1584713]
ENSRNOG00000011483	67.55	20.88	1.6882	0.001182	0.22857	*S100a9*	S100 calcium binding protein A9 [Source:RGD Symbol;Acc:620267]
ENSRNOG00000050193	41.28	13.02	1.6795	0.012264	0.84566	*LOC100912262*	general transcription factor II-I repeat domain-containing protein 1-like [Source:RGD Symbol;Acc:6488068]
Novel.10297	19.39	6.12	1.6609	0.009815	0.75177	-	-
Novel.2531	14.86	4.75	1.6406	0.024151	0.99978	-	-
Novel.1005	20.3	6.91	1.536	0.012302	0.84566	-	-
ENSRNOG00000030729	290.43	100.73	1.5268	5.33E-07	0.001114	*C4b*	complement C4B (Chido blood group) [Source:RGD Symbol;Acc:1591983]
ENSRNOG00000030387	63520.57	22084.34	1.5242	5.55E-15	5.79E-11	*Kng1*	kininogen 1 [Source:RGD Symbol;Acc:1359376]
Novel.5897	132.92	46.54	1.5075	2.79E-05	0.021647	-	-
Novel.3914	39159	14284.38	1.4549	0.00018	0.063761	-	-
ENSRNOG00000011381	31.45	11.52	1.4447	0.005152	0.55168	*Acsbg1*	acyl-CoA synthetase bubblegum family member 1 [Source:RGD Symbol;Acc:708557]
Novel.6054	18.85	6.9	1.4344	0.024385	0.99978	-	-
ENSRNOG00000057344	16.53	6.2	1.4282	0.037161	0.99978	*AABR07051733.2*	-
ENSRNOG00000060572	40.62	15.42	1.3955	0.002851	0.36996	*Tlk1*	tousled-like kinase 1 [Source:RGD Symbol;Acc:1310370]
Novel.3918	3027.82	1155.44	1.3897	1.34E-13	7.00E-10	-	-
ENSRNOG00000021027	594.85	229.33	1.374	0.02549	0.99978	*Dbp*	D-box binding PAR bZIP transcription factor [Source:RGD Symbol;Acc:2491]
ENSRNOG00000011557	25.69	9.97	1.3678	0.049715	0.99978	*S100a8*	S100 calcium binding protein A8 [Source:RGD Symbol;Acc:620265]
Novel.8685	35.02	13.59	1.3666	0.008688	0.70863	-	-

ENSRNOG00000049849	23.6	9.29	1.3533	0.015435	0.96766	*Fam199x*	family with sequence similarity 199, X-linked [Source:MGI Symbol;Acc:MGI:2384304]
ENSRNOG00000007886	18229.21	7264.35	1.3273	0.009412	0.73336	*Orm1*	orosomucoid 1 [Source:RGD Symbol;Acc:67390]
ENSRNOG00000011715	32.17	13.15	1.2832	0.010802	0.79705	*AABR07053669.1*	mitochondrial fission regulator 1, pseudo 1 [Source:NCBI gene;Acc:679959]
ENSRNOG00000009300	43.59	18.16	1.2677	0.036792	0.99978	*LOC103690017*	serine/threonine-protein kinase tousled-like 1 [Source:RGD Symbol;Acc:9355222]
Novel.8890	62.31	25.73	1.2619	0.002101	0.32253	-	-
ENSRNOG00000006663	82.86	35.12	1.2386	0.000123	0.050187	*Usp2*	ubiquitin specific peptidase 2 [Source:RGD Symbol;Acc:621073]
ENSRNOG00000029980	34.75	14.74	1.2385	0.028379	0.99978	*Zbtb16*	zinc finger and BTB domain containing 16 [Source:RGD Symbol;Acc:727921]
ENSRNOG00000029668	454.36	193.31	1.2337	0.008964	0.71718	*Wfdc21*	WAP four-disulfide core domain 21 [Source:RGD Symbol;Acc:1303147]
ENSRNOG00000033763	24.92	10.71	1.2104	0.021587	0.99978	*RGD1307537*	similar to RIKEN cDNA 4933417A18 [Source:RGD Symbol;Acc:1307537]
ENSRNOG00000018500	22.7	9.82	1.193	0.042013	0.99978	*AABR07028615.1*	-
ENSRNOG00000049751	40.84	18.39	1.1535	0.023693	0.99978	*Arid4b*	AT-rich interaction domain 4B [Source:RGD Symbol;Acc:619919]
ENSRNOG00000003884	102.54	46.28	1.1503	9.40E-05	0.044624	*Acmsd*	aminocarboxymuconate semialdehyde decarboxylase [Source:RGD Symbol;Acc:620868]
Novel.1417	102.81	46.65	1.1404	0.00016	0.061976	-	-
ENSRNOG00000021355	64.47	29.94	1.1177	0.002408	0.35255	*Car6*	carbonic anhydrase 6 [Source:RGD Symbol;Acc:70516]
ENSRNOG00000003947	40.28	18.49	1.1169	0.006942	0.6369	*Ntn1*	netrin 1 [Source:RGD Symbol;Acc:619809]
ENSRNOG00000059714	282.82	130.93	1.1157	0.00016	0.061976	*Hsp90aa1*	heat shock protein 90 alpha family class A member 1 [Source:RGD Symbol;Acc:631409]
ENSRNOG00000010714	27203.71	12687.06	1.1004	7.94E-11	3.32E-07	*Fgl1*	fibrinogen-like 1 [Source:RGD Symbol;Acc:620169]
ENSRNOG0000003134	37.43	17.85	1.0751	0.013612	0.88416	*Nxpe5*	neurexophilin and PC-esterase domain family,

3							member 5 [Source:RGD Symbol;Acc:1588255]
ENSRNOG00000013464	76.44	36.44	1.0671	0.004685	0.52598	*Spink1*	serine peptidase inhibitor, Kazal type 1 [Source:RGD Symbol;Acc:3749]
ENSRNOG00000008095	142.3	69.34	1.0425	7.47E-05	0.038999	*Onecut1*	one cut homeobox 1 [Source:RGD Symbol;Acc:2811]
ENSRNOG00000005479	325.69	159.29	1.0311	9.78E-07	0.001495	*Slc1a2*	solute carrier family 1 member 2 [Source:RGD Symbol;Acc:3697]
Novel.10596	653.84	322.94	1.0153	3.78E-06	0.003763	-	-
ENSRNOG00000003616	127.19	63.07	1.0135	4.03E-05	0.029049	*Grem2*	gremlin 2, DAN family BMP antagonist [Source:RGD Symbol;Acc:1560008]
ENSRNOG00000056688	44.05	89.63	-1.0176	0.006953	0.6369	*Acnat1*	acyl-coenzyme A amino acid N-acyltransferase 1 [Source:RGD Symbol;Acc:1584701]
ENSRNOG00000049053	21.95	44.58	-1.0275	0.012379	0.84566	*Glipr1l2*	GLIPR1 like 2 [Source:RGD Symbol;Acc:1310205]
Novel.1842	13.74	28.38	-1.0377	0.033408	0.99978	-	-
ENSRNOG00000051701	14.46	29.75	-1.0392	0.037341	0.99978	*AABR07073453.1*	-
ENSRNOG00000050910	61.33	126.54	-1.0438	0.003678	0.45173	*LOC688583*	similar to High mobility group protein 4 (HMG-4) (High mobility group protein 2a) (HMG-2a) [Source:RGD Symbol;Acc:1587596]
ENSRNOG00000000924	14.51	29.84	-1.0465	0.03227	0.99978	*Slc7a1*	solute carrier family 7 member 1 [Source:RGD Symbol;Acc:3716]
ENSRNOG00000007076	18.84	38.69	-1.0489	0.029335	0.99978	*AABR07055919.1*	-
Novel.15757	13.27	27.89	-1.056	0.039537	0.99978	-	-
Novel.16944	22.81	47.88	-1.0694	0.020857	0.99978	-	-
ENSRNOG00000007546	144.4	303.61	-1.0717	0.038343	0.99978	*Asns*	asparagine synthetase (glutamine-hydrolyzing) [Source:RGD Symbol;Acc:2162]
Novel.5477	11.77	24.89	-1.0812	0.04575	0.99978	-	-
ENSRNOG00000018029	13.62	28.92	-1.0832	0.02594	0.99978	*Doc2g*	double C2-like domains, gamma [Source:RGD Symbol;Acc:1307473]
Novel.16237	67.2	143.52	-1.0925	0.000112	0.049648	-	-
ENSRNOG0000001708	25.01	53.67	-1.0955	0.005741	0.58193	*Hsd11b2*	hydroxysteroid 11-beta dehydrogenase 2

4							[Source:RGD Symbol;Acc:2835]
ENSRNOG00000033130	261.85	580.99	-1.1502	0.01688	0.9865	*Mup4*	major urinary protein 4 [Source:RGD Symbol;Acc:735211]
ENSRNOG00000026764	11.75	26.6	-1.1723	0.025881	0.99978	*Pla2g4c*	phospholipase A2 group IVC [Source:RGD Symbol;Acc:1594658]
ENSRNOG00000006731	9.85	22.55	-1.1883	0.030028	0.99978	*Spc25*	SPC25, NDC80 kinetochore complex component [Source:RGD Symbol;Acc:1307811]
ENSRNOG00000024917	17.48	40.01	-1.1979	0.012688	0.85676	*Als2cr12*	amyotrophic lateral sclerosis 2 chromosome region 12 [Source:RGD Symbol;Acc:1359362]
ENSRNOG00000053301	19.31	44.71	-1.206	0.005078	0.54989	*LOC685767*	similar to OX-2 membrane glycoprotein precursor (MRC OX-2 antigen) (CD200 antigen) [Source:RGD Symbol;Acc:1590201]
ENSRNOG00000042918	13.27	31.02	-1.2151	0.013025	0.86617	*RGD1304770*	similar to Na+ dependent glucose transporter 1 [Source:RGD Symbol;Acc:1304770]
ENSRNOG00000028812	29.33	67.96	-1.2178	0.000222	0.074777	*Trim80*	tripartite motif protein 80 [Source:RGD Symbol;Acc:1311352]
Novel.12202	11.77	27.8	-1.2457	0.023503	0.99978	-	-
ENSRNOG00000028752	10.2	24.08	-1.2477	0.030893	0.99978	*Tmem81*	transmembrane protein 81 [Source:RGD Symbol;Acc:1561544]
ENSRNOG00000061749	13.81	33.29	-1.2557	0.01733	0.99969	*RF00100*	-
Novel.10356	30.13	72.29	-1.2564	0.000432	0.10747	-	-
ENSRNOG00000054371	7.78	18.79	-1.2772	0.03154	0.99978	*Prkcg*	protein kinase C, gamma [Source:RGD Symbol;Acc:3397]
Novel.13785	37.31	91.08	-1.2844	0.001258	0.23445	-	-
ENSRNOG00000030321	22.27	56.25	-1.3283	0.005888	0.58653	*LOC502176*	hypothetical protein LOC502176 [Source:RGD Symbol;Acc:1560919]
ENSRNOG00000010833	34.53	90.52	-1.3898	6.40E-05	0.036138	*Mthfd2*	methylenetetrahydrofolate dehydrogenase (NADP+ dependent) 2, methenyltetrahydrofolate cyclohydrolase [Source:RGD Symbol;Acc:1311313]
ENSRNOG00000008648	11.68	31.74	-1.453	0.04869	0.99978	*Mogs*	mannosyl-oligosaccharide glucosidase [Source:RGD Symbol;Acc:69240]

ENSRNOG00000057089	26.8	75.44	-1.4848	0.002906	0.37255	*LOC103691744*	cystathionine gamma-lyase [Source:RGD Symbol;Acc:9153842]
Novel.11553	4.87	13.87	-1.5063	0.038668	0.99978	-	-
ENSRNOG00000009596	17.63	49.57	-1.5069	0.005899	0.58653	*LOC103689945*	zinc finger SWIM domain-containing protein 8-like [Source:RGD Symbol;Acc:9188719]
ENSRNOG00000012481	6.81	19.39	-1.5159	0.016211	0.97716	*Ppm1j*	protein phosphatase, Mg2+/Mn2+ dependent, 1J [Source:RGD Symbol;Acc:1359104]
Novel.12679	8.12	23.55	-1.5373	0.005503	0.57455	-	-
Novel.11190	9.17	27.21	-1.5514	0.008849	0.7162	-	-
ENSRNOG00000046832	10.17	30.61	-1.5636	0.006634	0.6369	*AABR07039307.1*	-
ENSRNOG00000042721	25.98	77.64	-1.5666	2.65E-05	0.021647	*Gimd1*	GIMAP family P-loop NTPase domain containing 1 [Source:RGD Symbol;Acc:1563706]
Novel.5756	4.91	14.52	-1.5742	0.027442	0.99978	-	-
ENSRNOG00000013971	139.06	417.12	-1.5843	0.008887	0.7165	*Psat1*	phosphoserine aminotransferase 1 [Source:RGD Symbol;Acc:735170]
ENSRNOG00000015763	3.76	11.51	-1.5947	0.046809	0.99978	*Nat8f3*	N-acetyltransferase 8 (GCN5-related) family member 3 [Source:RGD Symbol;Acc:621607]
ENSRNOG00000052725	3.89	11.84	-1.6089	0.037103	0.99978	*Shisal1*	shisa like 1 [Source:RGD Symbol;Acc:1566029]
Novel.6392	6.56	20.48	-1.6446	0.009224	0.72753	-	-
ENSRNOG00000032740	5.16	16.22	-1.6455	0.016076	0.97716	*Tmem258b*	transmembrane protein 258 B [Source:RGD Symbol;Acc:1586604]
Novel.8463	3.98	12.46	-1.6645	0.042148	0.99978	-	-
ENSRNOG00000050240	4.23	13.4	-1.6717	0.02534	0.99978	*Mtf1*	metal-regulatory transcription factor 1 [Source:RGD Symbol;Acc:1308956]
ENSRNOG00000015818	12.83	41.3	-1.6809	0.005811	0.58341	*LOC103690067*	EKC/KEOPS complex subunit Tprkb [Source:RGD Symbol;Acc:9395731]
ENSRNOG00000009617	17.38	56.08	-1.7028	0.001611	0.26306	*LOC100911734*	exocyst complex component 7-like [Source:RGD Symbol;Acc:6491410]
Novel.13468	5.91	19.9	-1.7306	0.011765	0.82998	-	-
ENSRNOG00000009204	2.92	9.9	-1.7629	0.04981	0.99978	*Il17re*	interleukin 17 receptor E [Source:RGD Symbol;Acc:735148]

ENSRNOG00000051771	10.75	36.98	-1.7805	0.006796	0.6369	*AABR07006894.1*	-
Novel.15904	8.33	28.77	-1.7836	0.002853	0.36996	-	-
ENSRNOG00000001893	11.97	41.59	-1.7927	0.003966	0.46786	*LOC100362453*	serine/threonine-protein phosphatase 2A catalytic subunit alpha-like [Source:RGD Symbol;Acc:2318135]
ENSRNOG00000019328	58.93	208.68	-1.822	0.004835	0.53991	*Phgdh*	phosphoglycerate dehydrogenase [Source:RGD Symbol;Acc:61987]
ENSRNOG00000029830	5.37	19.25	-1.8342	0.00749	0.66833	*Adm2*	adrenomedullin 2 [Source:RGD Symbol;Acc:1302971]
Novel.1080	3.08	11.79	-1.9099	0.041281	0.99978	-	-
ENSRNOG00000049944	51.08	199.99	-1.9681	0.01669	0.983	*Slc25a22*	solute carrier family 25 member 22 [Source:RGD Symbol;Acc:1307826]
ENSRNOG00000016512	1.95	7.71	-1.9935	0.04897	0.99978	*Sema3b*	semaphorin 3B [Source:RGD Symbol;Acc:1310079]
ENSRNOG00000020990	130.21	522.48	-2.0034	6.98E-05	0.037381	*Fgf21*	fibroblast growth factor 21 [Source:RGD Symbol;Acc:620175]
ENSRNOG00000031060	4.48	18.92	-2.0657	0.008034	0.68391	*LOC498826*	LRRGT00165 [Source:RGD Symbol;Acc:1560045]
ENSRNOG00000024139	2.83	12.61	-2.128	0.015478	0.96766	*LOC100911456*	migration and invasion-inhibitory protein-like [Source:RGD Symbol;Acc:6492682]
ENSRNOG00000016278	2.58	11.53	-2.1506	0.017623	0.99978	*Ccl17*	C-C motif chemokine ligand 17 [Source:RGD Symbol;Acc:619924]
ENSRNOG00000059093	2.33	10.76	-2.2224	0.019982	0.99978	*AABR07012583.2*	-
Novel.16888	3.98	19.02	-2.2651	0.005663	0.5778	-	-
ENSRNOG00000028356	553.84	2892.59	-2.3848	0.00132	0.23965	*LOC103694872*	coiled-coil-helix-coiled-coil-helix domain-containing protein 10, mitochondrial [Source:RGD Symbol;Acc:9377836]
ENSRNOG00000050877	13.97	74.39	-2.4072	0.02695	0.99978	*LOC100912538*	centrin-3-like [Source:RGD Symbol;Acc:6486167]
ENSRNOG00000060990	3.51	18.91	-2.4161	0.010197	0.76865	*AABR07000382.1*	-

Novel.16891	2.24	12.17	-2.4367	0.026705	0.99978	-	-
ENSRNOG00000049575	9.89	58.13	-2.547	5.70E-05	0.035265	*Atn1*	atrophin 1 [Source:RGD Symbol;Acc:61832]
Novel.14753	1.7	9.72	-2.5499	0.0187	0.99978	-	-
ENSRNOG00000057327	1.27	7.57	-2.5578	0.029822	0.99978	*AABR07065118.1*	-
ENSRNOG00000030346	1.56	9.64	-2.5948	0.035952	0.99978	*AABR07038766.1*	similar to 60S ribosomal protein L32 [Source:NCBI gene;Acc:680959]
Novel.16887	9	56.9	-2.6607	0.010995	0.80554	-	-
ENSRNOG00000002824	1.65	10.63	-2.6986	0.026953	0.99978	*NEWGENE_1559832*	ring finger protein, LIM domain interacting [Source:RGD Symbol;Acc:11393862]
Novel.12728	1.61	10.86	-2.7444	0.00861	0.70863	-	-
ENSRNOG00000017606	1.02	7.25	-2.8707	0.026691	0.99978	*P2rx1*	purinergic receptor P2X 1 [Source:RGD Symbol;Acc:3240]
ENSRNOG00000029810	12.95	98.55	-2.926	1.67E-10	5.81E-07	*Tspan4*	tetraspanin 4 [Source:RGD Symbol;Acc:1305810]
ENSRNOG00000048701	0.68	6.17	-3.231	0.023457	0.99978	*Rpl38*	ribosomal protein L38 [Source:RGD Symbol;Acc:1305573]
ENSRNOG00000017156	0.59	6.4	-3.3504	0.021521	0.99978	*Ankle1*	ankyrin repeat and LEM domain containing 1 [Source:RGD Symbol;Acc:1308184]
ENSRNOG00000047971	7.06	72.82	-3.3642	0.002576	0.35891	*Mrpl36*	mitochondrial ribosomal protein L36 [Source:RGD Symbol;Acc:1306375]
ENSRNOG00000015428	8.12	89.55	-3.4665	2.60E-15	5.42E-11	*LOC102556337*	mitochondrial fission factor-like [Source:RGD Symbol;Acc:7706142]
ENSRNOG00000047812	3.17	43.38	-3.7694	0.007648	0.67669	*Triap1*	TP53 regulated inhibitor of apoptosis 1 [Source:RGD Symbol;Acc:1586233]
ENSRNOG00000058688	0.34	4.8	-3.784	0.022752	0.99978	*AABR07015517.2*	-
Novel.2187	0.88	13.98	-3.9001	0.003832	0.45982	-	-
ENSRNOG00000046192	1.27	19.48	-3.9245	0.001025	0.20196	*LOC103690007*	5-methylcytosine rRNA methyltransferase NSUN4 [Source:RGD Symbol;Acc:9205239]
ENSRNOG00000026087	0	3.12	-4.1226	0.034986	0.99978	*Igfn1*	immunoglobulin-like and fibronectin type III domain containing 1 [Source:RGD

							Symbol;Acc:1565384]
ENSRNOG00000042211	0	3.14	-4.1285	0.035947	0.99978	*Tmem240*	transmembrane protein 240 [Source:RGD Symbol;Acc:2318117]
ENSRNOG00000054326	0	3.56	-4.3239	0.035475	0.99978	*RF00604*	predicted gene, 23991 [Source:MGI Symbol;Acc:MGI:5453768]

Supplementary Table S4. Transcriptomics KEGG pathway

ID	Description	Test	TestAll	Ref	RefAll	Pvalue	FDR	richFactor	Gene_id
rno04657	IL-17 signaling pathway	5	42	94	8281	0.00010335	0.01601974	0.05319149	ENSRNOG00000011483丨ENSRNOG00000009204丨ENSRNOG00000011557丨ENSRNOG00000059714丨ENSRNOG00000016278
rno00260	Glycine, serine and threonine metabolism	3	42	40	8281	0.00105203	0.05069831	0.075	ENSRNOG00000013971丨ENSRNOG00000057089丨ENSRNOG00000019328
rno00920	Sulfur metabolism	2	42	12	8281	0.00160499	0.05069831	0.16666667	ENSRNOG00000053812丨ENSRNOG00000027079
rno01100	Metabolic pathways	16	42	1488	8281	0.00163543	0.05069831	0.01075269	ENSRNOG00000017084丨ENSRNOG00000007546丨ENSRNOG00000013971丨ENSRNOG00000026764丨ENSRNOG00000016173丨ENSRNOG00000015763丨ENSRNOG00000018736丨ENSRNOG00000003884丨ENSRNOG00000053812丨ENSRNOG00000011381丨ENSRNOG00000057089丨ENSRNOG00000027079丨ENSRNOG00000008648丨ENSRNOG00000010833丨ENSRNOG00000021355丨ENSRNOG00000019328
rno01230	Biosynthesis of amino acids	4	42	82	8281	0.00075089	0.05069831	0.04878049	ENSRNOG00000007546丨ENSRNOG00000013971丨ENSRNOG00000057089丨ENSRNOG00000019328
rno00270	Cysteine and methionine metabolism	3	42	50	8281	0.00201486	0.05205067	0.06	ENSRNOG00000013971丨ENSRNOG00000057089丨ENSRNOG00000019328
rno00591	Linoleic acid metabolism	2	42	37	8281	0.01494929	0.27876957	0.05405405	ENSRNOG00000026764丨ENSRNOG00000016173
rno04750	Inflammatory mediator regulation of TRP channels	3	42	110	8281	0.01798513	0.27876957	0.02727273	ENSRNOG00000026764丨ENSRNOG00000030387丨ENSRNOG00000054371
rno04960	Aldosterone-regulated sodium reabsorption	2	42	39	8281	0.01652701	0.27876957	0.05128205	ENSRNOG00000017084丨ENSRNOG00000054371
rno05143	African trypanosomiasis	2	42	38	8281	0.01572955	0.27876957	0.05263158	ENSRNOG00000030387丨ENSRNOG00000054371

rno04724	Glutamatergic synapse	3	42	115	8281	0.02022069	0.28492785	0.02608696	ENSRNOG00000026764丨ENSRNOG00000006957丨ENSRNOG00000054371
rno00232	Caffeine metabolism	1	42	6	8281	0.03005681	0.30126703	0.16666667	ENSRNOG00000016173
rno00380	Tryptophan metabolism	2	42	48	8281	0.02444791	0.30126703	0.04166667	ENSRNOG00000016173丨ENSRNOG00000003884
rno04370	VEGF signaling pathway	2	42	57	8281	0.03361707	0.30126703	0.03508772	ENSRNOG00000026764丨ENSRNOG00000054371
rno04730	Long-term depression	2	42	60	8281	0.03692951	0.30126703	0.03333333	ENSRNOG00000026764丨ENSRNOG00000054371
rno04940	Type I diabetes mellitus	2	42	60	8281	0.03692951	0.30126703	0.03333333	ENSRNOG00000051002丨ENSRNOG00000045973
rno05320	Autoimmune thyroid disease	2	42	60	8281	0.03692951	0.30126703	0.03333333	ENSRNOG00000051002丨ENSRNOG00000045973
rno05330	Allograft rejection	2	42	53	8281	0.02939579	0.30126703	0.03773585	ENSRNOG00000051002丨ENSRNOG00000045973
rno05332	Graft-versus-host disease	2	42	51	8281	0.0273718	0.30126703	0.03921569	ENSRNOG00000051002丨ENSRNOG00000045973
rno00140	Steroid hormone biosynthesis	2	42	64	8281	0.04153317	0.32188205	0.03125	ENSRNOG00000017084丨ENSRNOG00000016173
rno03010	Ribosome	3	42	159	8281	0.0462241	0.3256698	0.01886792	ENSRNOG00000048701丨ENSRNOG00000047971丨ENSRNOG00000007663
rno05031	Amphetamine addiction	2	42	67	8281	0.04512077	0.3256698	0.02985075	ENSRNOG00000006957丨ENSRNOG00000054371
rno00750	Vitamin B6 metabolism	1	42	10	8281	0.04960281	0.33427983	0.1	ENSRNOG00000013971
rno00061	Fatty acid biosynthesis	1	42	18	8281	0.08754813	0.37887737	0.05555556	ENSRNOG00000011381
rno00670	One carbon pool by folate	1	42	18	8281	0.08754813	0.37887737	0.05555556	ENSRNOG00000010833
rno00910	Nitrogen metabolism	1	42	18	8281	0.08754813	0.37887737	0.05555556	ENSRNOG00000021355
rno01521	EGFR tyrosine kinase inhibitor resistance	2	42	79	8281	0.0605418	0.37887737	0.02531646	ENSRNOG00000007663丨ENSRNOG00000054371
rno04070	Phosphatidylinositol signaling system	2	42	95	8281	0.08344394	0.37887737	0.02105263	ENSRNOG00000027079丨ENSRNOG00000054371
rno04350	TGF-beta signaling pathway	2	42	95	8281	0.08344394	0.37887737	0.02105263	ENSRNOG00000003616丨ENSRNOG00000000556

rno04610	Complement and coagulation cascades	2	42	84	8281	0.06743232	0.37887737	0.02380952	ENSRNOG00000047657丨ENSRNOG00000030387
rno04612	Antigen processing and presentation	2	42	80	8281	0.06189922	0.37887737	0.025	ENSRNOG00000051002丨ENSRNOG00000059714
rno04650	Natural killer cell mediated cytotoxicity	2	42	92	8281	0.07896756	0.37887737	0.02173913	ENSRNOG00000045973丨ENSRNOG00000054371
rno04666	Fc gamma R-mediated phagocytosis	2	42	91	8281	0.07749311	0.37887737	0.02197802	ENSRNOG00000026764丨ENSRNOG00000054371
rno04713	Circadian entrainment	2	42	98	8281	0.08799733	0.37887737	0.02040816	ENSRNOG00000006957丨ENSRNOG00000054371
rno05150	Staphylococcus aureus infection	2	42	91	8281	0.07749311	0.37887737	0.02197802	ENSRNOG00000047657丨ENSRNOG00000051002
rno05231	Choline metabolism in cancer	2	42	95	8281	0.08344394	0.37887737	0.02105263	ENSRNOG00000026764丨ENSRNOG00000054371
rno00450	Selenocompound metabolism	1	42	19	8281	0.09218603	0.38618472	0.05263158	ENSRNOG00000057089
rno04659	Th17 cell differentiation	2	42	103	8281	0.09574868	0.39055383	0.01941748	ENSRNOG00000051002丨ENSRNOG00000059714
rno00592	alpha-Linolenic acid metabolism	1	42	23	8281	0.11050859	0.40638463	0.04347826	ENSRNOG00000026764
rno01200	Carbon metabolism	2	42	120	8281	0.12344621	0.40638463	0.01666667	ENSRNOG00000013971丨ENSRNOG00000019328
rno04014	Ras signaling pathway	3	42	231	8281	0.11142287	0.40638463	0.01298701	ENSRNOG00000026764丨ENSRNOG00000020990丨ENSRNOG00000054371
rno04066	HIF-1 signaling pathway	2	42	110	8281	0.10691748	0.40638463	0.01818182	ENSRNOG00000007663丨ENSRNOG00000054371
rno04071	Sphingolipid signaling pathway	2	42	121	8281	0.12513274	0.40638463	0.01652893	ENSRNOG00000030387丨ENSRNOG00000054371
rno04714	Thermogenesis	3	42	234	8281	0.11466489	0.40638463	0.01282051	ENSRNOG00000018736丨ENSRNOG00000007663丨ENSRNOG00000020990
rno04726	Serotonergic synapse	2	42	121	8281	0.12513274	0.40638463	0.01652893	ENSRNOG00000026764丨ENSRNOG00000054371
rno04950	Maturity onset diabetes of	1	42	27	8281	0.12846998	0.40638463	0.03703704	ENSRNOG00000008095

	the young								
rno05200	Pathways in cancer	5	42	528	8281	0.12676637	0.40638463	0.0094697	ENSRNOG00000030387丨ENSRNOG00000029980丨ENSRNOG00000059714丨ENSRNOG00000020990丨ENSRNOG00000054371
rno05310	Asthma	1	42	27	8281	0.12846998	0.40638463	0.03703704	ENSRNOG00000051002
rno05322	Systemic lupus erythematosus	2	42	116	8281	0.11675926	0.40638463	0.01724138	ENSRNOG00000047657丨ENSRNOG00000051002
rno04611	Platelet activation	2	42	126	8281	0.13364922	0.41431257	0.01587302	ENSRNOG00000026764丨ENSRNOG00000017606
rno04270	Vascular smooth muscle contraction	2	42	132	8281	0.14404087	0.42125161	0.01515152	ENSRNOG00000026764丨ENSRNOG00000054371
rno04728	Dopaminergic synapse	2	42	131	8281	0.14229668	0.42125161	0.01526718	ENSRNOG00000006957丨ENSRNOG00000054371
rno04910	Insulin signaling pathway	2	42	129	8281	0.13882273	0.42125161	0.01550388	ENSRNOG00000009617丨ENSRNOG00000007663
rno04060	Cytokine-cytokine receptor interaction	3	42	265	8281	0.15017173	0.43104848	0.01132075	ENSRNOG00000009204丨ENSRNOG00000000556丨ENSRNOG00000016278
rno04550	Signaling pathways regulating pluripotency of stem cells	2	42	139	8281	0.15637652	0.44069747	0.01438849	ENSRNOG00000000556丨ENSRNOG00000008095
rno00250	Alanine, aspartate and glutamate metabolism	1	42	36	8281	0.16759833	0.46388824	0.02777778	ENSRNOG00000007546
rno04217	Necroptosis	2	42	147	8281	0.17071767	0.46423226	0.01360544	ENSRNOG00000026764丨ENSRNOG00000059714
rno04010	MAPK signaling pathway	3	42	295	8281	0.18750372	0.4687593	0.01016949	ENSRNOG00000026764丨ENSRNOG00000020990丨ENSRNOG00000054371
rno04150	mTOR signaling pathway	2	42	155	8281	0.18527836	0.4687593	0.01290323	ENSRNOG00000007663丨ENSRNOG00000054371
rno04723	Retrograde endocannabinoid signaling	2	42	154	8281	0.18344763	0.4687593	0.01298701	ENSRNOG00000006957丨ENSRNOG00000054371
rno04921	Oxytocin signaling pathway	2	42	152	8281	0.17979491	0.4687593	0.01315789	ENSRNOG00000026764丨ENSRNOG00000054371

rno05033	Nicotine addiction	1	42	40	8281	0.18443321	0.4687593	0.025	ENSRNOG00000006957
rno00071	Fatty acid degradation	1	42	44	8281	0.20093557	0.47189414	0.02272727	ENSRNOG00000011381
rno03022	Basal transcription factors	1	42	42	8281	0.19272555	0.47189414	0.02380952	ENSRNOG00000050193
rno04141	Protein processing in endoplasmic reticulum	2	42	163	8281	0.2000196	0.47189414	0.01226994	ENSRNOG00000059714|ENSRNOG00000008648
rno04672	Intestinal immune network for IgA production	1	42	43	8281	0.1968408	0.47189414	0.02325581	ENSRNOG00000051002
rno04022	cGMP-PKG signaling pathway	2	42	166	8281	0.20558661	0.47561081	0.01204819	ENSRNOG00000030387|ENSRNOG00000050193
rno00565	Ether lipid metabolism	1	42	46	8281	0.20906406	0.47654307	0.02173913	ENSRNOG00000026764
rno00480	Glutathione metabolism	1	42	64	8281	0.2786796	0.47890257	0.015625	ENSRNOG00000015763
rno00510	N-Glycan biosynthesis	1	42	50	8281	0.2250796	0.47890257	0.02	ENSRNOG00000008648
rno00561	Glycerolipid metabolism	1	42	61	8281	0.26750753	0.47890257	0.01639344	ENSRNOG00000018736
rno00562	Inositol phosphate metabolism	1	42	75	8281	0.31823848	0.47890257	0.01333333	ENSRNOG00000027079
rno00830	Retinol metabolism	1	42	64	8281	0.2786796	0.47890257	0.015625	ENSRNOG00000016173
rno00980	Metabolism of xenobiotics by cytochrome P450	1	42	56	8281	0.24851096	0.47890257	0.01785714	ENSRNOG00000016173
rno00982	Drug metabolism - cytochrome P450	1	42	56	8281	0.24851096	0.47890257	0.01785714	ENSRNOG00000016173
rno01212	Fatty acid metabolism	1	42	60	8281	0.26374611	0.47890257	0.01666667	ENSRNOG00000011381
rno04015	Rap1 signaling pathway	2	42	211	8281	0.2903883	0.47890257	0.00947867	ENSRNOG00000020990|ENSRNOG00000054371
rno04020	Calcium signaling pathway	2	42	192	8281	0.25442354	0.47890257	0.01041667	ENSRNOG00000017606|ENSRNOG00000054371
rno04080	Neuroactive ligand-receptor interaction	3	42	347	8281	0.25704938	0.47890257	0.00864553	ENSRNOG00000030387|ENSRNOG00000006957|ENSRNOG00000017606
rno04151	PI3K-Akt signaling pathway	3	42	340	8281	0.24742866	0.47890257	0.00882353	ENSRNOG00000007663|ENSRNOG00000059714|ENSRNOG00000020990
rno04360	Axon guidance	2	42	180	8281	0.23178132	0.47890257	0.01111111	ENSRNOG00000003947|ENSRNOG00000016512
rno04664	Fc epsilon RI signaling	1	42	66	8281	0.28603505	0.47890257	0.01515152	ENSRNOG00000026764

	pathway								
rno04720	Long-term potentiation	1	42	63	8281	0.27497419	0.47890257	0.01587302	ENSRNOG00000054371
rno04810	Regulation of actin cytoskeleton	2	42	213	8281	0.29417085	0.47890257	0.00938967	ENSRNOG00000030387|ENSRNOG00000020990
rno04913	Ovarian steroidogenesis	1	42	51	8281	0.22903375	0.47890257	0.01960784	ENSRNOG00000026764
rno04918	Thyroid hormone synthesis	1	42	73	8281	0.31120704	0.47890257	0.01369863	ENSRNOG00000054371
rno04920	Adipocytokine signaling pathway	1	42	70	8281	0.30052695	0.47890257	0.01428571	ENSRNOG00000011381
rno04923	Regulation of lipolysis in adipocytes	1	42	57	8281	0.25234835	0.47890257	0.01754386	ENSRNOG00000018736
rno04929	GnRH secretion	1	42	64	8281	0.2786796	0.47890257	0.015625	ENSRNOG00000054371
rno04961	Endocrine and other factor-regulated calcium reabsorption	1	42	59	8281	0.25996583	0.47890257	0.01694915	ENSRNOG00000054371
rno04970	Salivary secretion	1	42	75	8281	0.31823848	0.47890257	0.01333333	ENSRNOG00000054371
rno04971	Gastric acid secretion	1	42	74	8281	0.31473156	0.47890257	0.01351351	ENSRNOG00000054371
rno05133	Pertussis	1	42	70	8281	0.30052695	0.47890257	0.01428571	ENSRNOG00000047657
rno05140	Leishmaniasis	1	42	70	8281	0.30052695	0.47890257	0.01428571	ENSRNOG00000051002
rno05202	Transcriptional misregulation in cancer	2	42	184	8281	0.2393149	0.47890257	0.01086957	ENSRNOG00000045973|ENSRNOG00000029980
rno05204	Chemical carcinogenesis	1	42	74	8281	0.31473156	0.47890257	0.01351351	ENSRNOG00000016173
rno05205	Proteoglycans in cancer	2	42	208	8281	0.28471148	0.47890257	0.00961538	ENSRNOG00000007663|ENSRNOG00000054371
rno05214	Glioma	1	42	72	8281	0.30766482	0.47890257	0.01388889	ENSRNOG00000054371
rno05218	Melanoma	1	42	71	8281	0.30410482	0.47890257	0.01408451	ENSRNOG00000020990
rno05221	Acute myeloid leukemia	1	42	68	8281	0.29331726	0.47890257	0.01470588	ENSRNOG00000029980
rno05223	Non-small cell lung cancer	1	42	65	8281	0.28236652	0.47890257	0.01538462	ENSRNOG00000054371
rno05321	Inflammatory bowel disease (IBD)	1	42	61	8281	0.26750753	0.47890257	0.01639344	ENSRNOG00000051002
rno05416	Viral myocarditis	1	42	75	8281	0.31823848	0.47890257	0.01333333	ENSRNOG00000051002
rno00590	Arachidonic acid metabolism	1	42	77	8281	0.32519984	0.48467283	0.01298701	ENSRNOG00000026764

rno03320	PPAR signaling pathway	1	42	81	8281	0.33891501	0.5003031	0.01234568	ENSRNOG00000011381
rno04012	ErbB signaling pathway	1	42	86	8281	0.35567673	0.50134836	0.01162791	ENSRNOG00000054371
rno04061	Viral protein interaction with cytokine and cytokine receptor	1	42	84	8281	0.34902243	0.50134836	0.01190476	ENSRNOG00000016278
rno04540	Gap junction	1	42	88	8281	0.36226462	0.50134836	0.01136364	ENSRNOG00000054371
rno04640	Hematopoietic cell lineage	1	42	88	8281	0.36226462	0.50134836	0.01136364	ENSRNOG00000051002
rno04658	Th1 and Th2 cell differentiation	1	42	87	8281	0.35897894	0.50134836	0.01149425	ENSRNOG00000051002
rno04911	Insulin secretion	1	42	86	8281	0.35567673	0.50134836	0.01162791	ENSRNOG00000054371
rno05323	Rheumatoid arthritis	1	42	88	8281	0.36226462	0.50134836	0.01136364	ENSRNOG00000051002
rno04727	GABAergic synapse	1	42	91	8281	0.37202333	0.50142276	0.01098901	ENSRNOG00000054371
rno04912	GnRH signaling pathway	1	42	91	8281	0.37202333	0.50142276	0.01098901	ENSRNOG00000026764
rno04914	Progesterone-mediated oocyte maturation	1	42	90	8281	0.36878674	0.50142276	0.01111111	ENSRNOG00000059714
rno00564	Glycerophospholipid metabolism	1	42	97	8281	0.39110541	0.50481646	0.01030928	ENSRNOG00000026764
rno04916	Melanogenesis	1	42	98	8281	0.39423024	0.50481646	0.01020408	ENSRNOG00000054371
rno04925	Aldosterone synthesis and secretion	1	42	95	8281	0.38480848	0.50481646	0.01052632	ENSRNOG00000054371
rno05032	Morphine addiction	1	42	94	8281	0.38163622	0.50481646	0.0106383	ENSRNOG00000054371
rno05146	Amoebiasis	1	42	99	8281	0.3973394	0.50481646	0.01010101	ENSRNOG00000054371
rno05206	MicroRNAs in cancer	2	42	264	8281	0.38911562	0.50481646	0.00757576	ENSRNOG00000054371|ENSRNOG00000000924
rno05215	Prostate cancer	1	42	98	8281	0.39423024	0.50481646	0.01020408	ENSRNOG00000059714
rno04972	Pancreatic secretion	1	42	103	8281	0.40962104	0.5120263	0.00970874	ENSRNOG00000054371
rno05142	Chagas disease (American trypanosomiasis)	1	42	103	8281	0.40962104	0.5120263	0.00970874	ENSRNOG00000030387
rno04928	Parathyroid hormone synthesis, secretion and action	1	42	106	8281	0.41867159	0.51915277	0.00943396	ENSRNOG00000054371
rno04625	C-type lectin receptor signaling pathway	1	42	108	8281	0.42462989	0.52236216	0.00925926	ENSRNOG00000016278

rno05145	Toxoplasmosis	1	42	110	8281	0.43052856	0.52544824	0.00909091	ENSRNOG00000051002
rno04670	Leukocyte transendothelial migration	1	42	113	8281	0.43926604	0.52654821	0.00884956	ENSRNOG00000054371
rno04725	Cholinergic synapse	1	42	113	8281	0.43926604	0.52654821	0.00884956	ENSRNOG00000054371
rno04919	Thyroid hormone signaling pathway	1	42	115	8281	0.44501817	0.52654821	0.00869565	ENSRNOG00000054371
rno04935	Growth hormone synthesis, secretion and action	1	42	114	8281	0.44214934	0.52654821	0.00877193	ENSRNOG00000054371
rno03040	Spliceosome	1	42	132	8281	0.49164103	0.57296512	0.00757576	ENSRNOG00000032232
rno04915	Estrogen signaling pathway	1	42	132	8281	0.49164103	0.57296512	0.00757576	ENSRNOG00000059714
rno04210	Apoptosis	1	42	134	8281	0.49686802	0.5747354	0.00746269	ENSRNOG00000045973
rno04371	Apelin signaling pathway	1	42	138	8281	0.50716508	0.58230064	0.00724638	ENSRNOG00000007663
rno05224	Breast cancer	1	42	147	8281	0.52958703	0.59482601	0.00680272	ENSRNOG00000020990
rno05226	Gastric cancer	1	42	147	8281	0.52958703	0.59482601	0.00680272	ENSRNOG00000020990
rno05418	Fluid shear stress and atherosclerosis	1	42	147	8281	0.52958703	0.59482601	0.00680272	ENSRNOG00000059714
rno04072	Phospholipase D signaling pathway	1	42	150	8281	0.5368373	0.59863152	0.00666667	ENSRNOG00000026764
rno04310	Wnt signaling pathway	1	42	156	8281	0.55101208	0.60145684	0.00641026	ENSRNOG00000054371
rno04514	Cell adhesion molecules (CAMs)	1	42	156	8281	0.55101208	0.60145684	0.00641026	ENSRNOG00000051002
rno05161	Hepatitis B	1	42	154	8281	0.54633486	0.60145684	0.00649351	ENSRNOG00000054371
rno05164	Influenza A	1	42	158	8281	0.55564221	0.60226952	0.00632911	ENSRNOG00000051002
rno04145	Phagosome	1	42	169	8281	0.58028674	0.61605784	0.00591716	ENSRNOG00000051002
rno04621	NOD-like receptor signaling pathway	1	42	168	8281	0.57810263	0.61605784	0.00595238	ENSRNOG00000059714
rno05152	Tuberculosis	1	42	168	8281	0.57810263	0.61605784	0.00595238	ENSRNOG00000051002
rno05225	Hepatocellular carcinoma	1	42	174	8281	0.59104279	0.62320838	0.00574713	ENSRNOG00000054371
rno04062	Chemokine signaling pathway	1	42	176	8281	0.59526945	0.62342408	0.00568182	ENSRNOG00000016278

rno04510	Focal adhesion	1	42	200	8281	0.64277966	0.66866341	0.005	ENSRNOG00000054371
rno04024	cAMP signaling pathway	1	42	211	8281	0.66269183	0.68204859	0.00473934	ENSRNOG00000006957
rno05169	Epstein-Barr virus infection	1	42	212	8281	0.66444734	0.68204859	0.00471698	ENSRNOG00000051002
rno05170	Human immunodeficiency virus 1 infection	1	42	219	8281	0.67648859	0.68984033	0.00456621	ENSRNOG00000054371
rno05166	Human T-cell leukemia virus 1 infection	1	42	231	8281	0.69615656	0.70525665	0.004329	ENSRNOG00000051002
rno05163	Human cytomegalovirus infection	1	42	236	8281	0.70400256	0.70857401	0.00423729	ENSRNOG00000054371
rno05168	Herpes simplex virus 1 infection	1	42	321	8281	0.81074615	0.81074615	0.00311526	ENSRNOG00000051002
